# Drinking Pattern, Abstention and Problem Drinking as Risk Factors for Depressive Symptoms: Evidence from Three Urban Eastern European Populations

**DOI:** 10.1371/journal.pone.0104384

**Published:** 2014-08-13

**Authors:** Steven Bell, Annie Britton, Ruzena Kubinova, Sofia Malyutina, Andrzej Pajak, Yuri Nikitin, Martin Bobak

**Affiliations:** 1 Research Department of Epidemiology and Public Health, University College London, London, United Kingdom; 2 National Institute of Public Health, Prague, Czech Republic; 3 Institute of Internal and Preventive Medicine, Novosibirsk, Russia; 4 Novosibirsk State Medical University, Novosibirsk, Russia; 5 Institute of Public Health, Jagiellonian University, Krakow, Poland; University of Granada, Spain

## Abstract

**Purpose:**

To examine whether the frequency and amount of alcohol consumed in binge drinking sessions, total annual volume of alcohol consumed, problem drinking and abstaining from alcohol are associated with depressive symptoms in Eastern Europe.

**Subjects and Methods:**

Cross-sectional data from a total of 24,381 participants from general population samples of the Czech Republic (N = 7,601), Russia (N = 6,908) and Poland (N = 9,872) aged 45–69 years in 2002–2005. Depressive symptoms were defined as ≥16 points on the Centre for Epidemiological Studies – Depression (CES-D) scale. Several alcohol related measures were derived using responses from the graduated frequency questionnaire. Binge drinking was defined at several sex-specific thresholds (ranging from 60+ to 140+ g of ethanol) and two frequencies (at least monthly or weekly). Total annual alcohol intake in grams was also extracted. Problem drinking was defined as ≥2 positive answers on the CAGE questionnaire.

**Results:**

Problem drinking was consistently associated with approximately a 2-fold increase in odds of depressive symptoms across all countries and in both sexes. Abstaining from alcohol was typically associated with increased odds of depressive symptoms. Analyses separating lifelong abstainers and former drinkers in the Russian cohort revealed that this increased odds was driven by former drinkers. Amongst men, heavy frequent binge drinking was associated with increased odds of depressive symptoms in the Czech Republic and Poland. In women, heavy infrequent binge drinking was associated with increased odds of depressive symptoms in Russia and Poland. Only in Polish men was higher annual volume of alcohol intake associated with increased odds of depressive symptoms.

**Conclusion:**

Abstaining from alcohol and problem drinking were associated with increased odds of depressive symptoms in these Eastern European populations. Annual volume of alcohol intake as well as frequency and amount of alcohol consumed in a binge drinking session were less consistently associated with depressive symptoms.

## Introduction

Depression [1]–[3] and alcohol consumption [4] are widely acknowledged as important public health problems and there is ample evidence that the two are related [5], [6]. The majority of research on the topic has focussed on the relationship between clinical disorders [7]–[9] with less attention drawn to the association between alcohol intake and depressive symptoms in the general (non-clinical) population [10], [11]. Studies that have looked at the relationship between alcohol intake and depressive symptoms have produced conflicting findings [10]–[14].

Both the prevalence of depression [15], [16] and alcohol consumption [4] are high in Eastern Europe compared to other regions. Little is known about the extent to which drinking patterns in this region, marked by heavy alcohol consumption and binge drinking [4], [17], are linked with the high prevalence of depression. Examining the association between drinking pattern and depressive symptoms in populations with both high overall volume of alcohol intake combined with a pattern of heavy episodic drinking would allow for comparisons to be made to other drinking cultures whereby overall drinking volume is similar but drinking pattern is different (for example, Southern Europe [4]) or where drinking pattern is similar but overall volume of intake is lower (e.g. the Nordic regions [4]) to determine the extent to which drinking pattern plays a role in the development of depressive symptoms or whether it is mostly due to overall volume consumed.

Differentiating between different patterns of heavy drinking has been particularly encouraged in the context of Eastern Europe where a high prevalence of episodic heavy drinking exists [18]–[20]. Yet little research has specifically focussed on the effect of increasing dose of alcohol consumed per occasion *and* frequency of consuming these different doses of alcohol as risk factors for depression. It is probable that the biological effect of alcohol on the brain/body depends on cumulative dose, therefore examining the effect of different doses of consumption as well as the frequency of drinking makes intuitive sense. Others have, however, argued that it is problem drinking which is harmful to psychological health [21], but surprisingly few studies have examined the relationship between problem drinking and depression in Eastern Europe [22], [23]. On the other spectrum of drinking, the extent to which non-drinking is associated with depression is also underexplored. The main uncertainty here relates to the degree to which the supposed beneficial psychological effects of moderate consumption [24], [25] may be confounded by including former drinkers alongside lifelong abstainers in the non-drinking group [26], [27].

Given the paucity of research on these important public health issues, the purpose of this study was to determine the extent to which binge drinking at different intensities (i.e. doses) and frequencies (e.g. on a monthly or weekly basis), annual drinking volume, problem drinking and abstention from alcohol are related to depressive symptoms in Eastern Europe.

## Materials and Methods

### Ethical statement

The University College London Hospital Research Ethics Committee Alpha approved the HAPIEE study. Ethical approval was also obtained from local research ethics committees at each HAPIEE site: National Institute of Public Health Ethics Committee (Prague, the Czech Republic), Jagellonian University Medical College Bioethics Committee (Krakow, Poland), and Institute of Internal and Preventive Medicine Ethics Committee (Novosibirsk, Russia). Participants provided written, informed consent.

### The HAPIEE Study

Cross-sectional data from the baseline wave (2003–2005) of the Health, Alcohol and Psychosocial factors in Eastern Europe (HAPIEE) study [28] were used to explore the relationship between drinking pattern, problem drinking and abstaining from alcohol as risk factors for depressive symptoms. The study randomly recruited individuals aged between 45 to 69 years from electoral lists in Novosibirsk (Russia), and population registers in Krakow (Poland), Havirov/Karvina, Hradec Kralove, Jihlava, Kromeriz, Liberec and Usti nad Labem (Czech Republic). Questionnaires were identical across study sites and were translated to each language and back-translated to English to ensure comparability. The only difference between centres was the fact that the Polish questionnaire also included a filter question allowing those who reported not drinking in the previous 12 months to skip the other questions in the alcohol section. A total of 28,947 subjects (overall response rate of 59%) were recruited. The analytic sample consisted of 24, 381 individuals after those with missing information on depressive symptom status (N = 3,274), alcohol consumption (N = 376) and covariates (N = 509) were excluded (categories not mutually exclusive). A multivariable logistic regression model revealed that those with missing data were more likely to have lower educational attainment and belong to the high deprivation group.

### Assessment of dose and frequency of binge drinking

Data on drinking patterns come from the graduated frequency questionnaire [29], [30]. The graduated frequency questionnaire asks participants how often in the previous 12 months they consumed more than *X* amount of alcohol with separate doses, equivalent to: ≥10 g, ≥40 g, ≥60 g, ≥100 g, ≥140 g and ≥180 g of pure ethanol (based on original local units defined as 0.5 litre of beer, 0.2 litre of wine and 0.05 litre of spirits, ranging from “10 and above” to “less than one” - 100 ml of beer, wine and spirit was assumed to contain 4 g, 10 g, or 36 g of ethanol, respectively). There were nine frequency responses, spanning from never to daily.

Given gender differences in alcohol consumption and metabolism [31], combined with low cell counts for certain response categories in women, separate binge drinking variables were derived for men and women. In men it was coded as follows: (1, reference category) always consuming between 1–59 g of alcohol per single drinking occasion, (2) consuming 60+ g on an occasional basis (defined as less than monthly), (3) 60–99 g in a single sitting at least monthly, (4) 100–139 g in a single sitting at least monthly, (5) 140+g in a single sitting at least monthly, (6) 60–99 g in a single sitting at least weekly, (7) 100–139 g in a single sitting at least weekly, (8) 140+g in a single sitting at least weekly and (9) past-year abstainers (those who reported no alcohol consumption in the previous 12 months on the graduated frequency questionnaire). For women, categories 4 and 5, and 7 and 8 were combined to form 100+g on an at least monthly or weekly basis respectively. We used frequency to determine the category drinkers belonged to in cases whereby participants reported exceeding one threshold on a monthly basis and another on a weekly basis (e.g. if a participant consumed 140+ g on a monthly basis and 60–99 g on a weekly basis then they would be classified as belonging to the 60–99 g on a weekly basis group). The rationale behind this decision is that frequency better reflects usual drinking habits than overall amount alone. Approximately 18 and 11% of men categorised as belonging to the 60–99 g in a single sitting on a weekly basis category drank 100 g or 140 g in a single occasion on a monthly basis, respectively. Around 19% of men belonging to the 100–139 g on a weekly basis category drank 140+ g in a single drinking episode on a monthly basis. Almost 13% of women belonging to the category of 60–99 g in a single sitting on a weekly basis drank 100+ g on one occasion monthly.

The purpose of deriving this variable was to compare each dose/frequency category (as well as abstention) with a non-binge drinking reference group always consuming between 1 and <59 g of alcohol per occasion; not simply, for example, looking at those who drank ≥100 grams in a single sitting on a monthly basis compared to those who did not consume this amount (a category which would then also include participants who were themselves binge drinkers albeit at a lower threshold). However, for the sake of completeness, we also estimated models examining the relationship between frequency of consuming 60+, 100+ and 140+g and depressive symptoms separately. For each dose of alcohol, frequency responses were as follows: (1) never, (2) 1–11 times per year, (3) 1–3 times per month, (4) weekly, and (5) past year abstainers. At doses of 100+ and 140+ g per occasion the category of never was divided into those always consuming between 1–59 g of alcohol per single drinking occasion (referent) and those who drank above 60 g but always less than *X* g per occasion. The estimates from these models can be found in *Information S1*.

### Drinking volume

From the graduated frequency we also derived a measure of total volume of alcohol consumed in the previous year. We categorised consumption levels above 240 g (a threshold reflecting less than one drink per month) into sex-specific tertiles of consumption derived from the entire sample. These were 260–1740 g, 1750–6020 g and >6400 g for men, and 260–420 g, 430–1200 g and >1210 g for women. The final variable used in analyses consisted of the following categories: (1) abstainers, (2) ≤240 g per annum, (3) first tertile of consumption, (4) second tertile of consumption (reference category), and (5) third tertile of consumption.

### Problem drinking

Problem drinking was assessed using the CAGE questionnaire [32], [33]. CAGE is an acronym of the first four letters of the key words in each question: 1) “Have you ever felt you should Cut down on your drinking?”, 2) “Have people ever Annoyed you by criticising your drinking?”, 3) “Have you ever felt bad or Guilty about your drinking?”, and 4) “Have you ever had a drink first thing in the morning to steady your nerves or get rid of a hangover (as an “Eye opener”)?”. Positive responses to two or more of the questions are commonly used as an indicator of problem drinking [34].

### Former drinkers and lifelong abstainers

In the Russian cohort those who reported having not consumed alcohol in the past 12 months were asked whether they had previously consumed alcohol. These questions were used to stratify the abstainer group into former drinkers and life-long abstainers.

### Assessment of depressive symptoms

Depressive symptoms were assessed using the 20-item Center for Epidemiologic Studies Depression (CES-D) scale [35]. The CES-D is a self-report questionnaire aimed to measure the presence of depressive symptoms in the past week (e.g. “I felt depressed” or “I was bothered by things that usually don't bother me”). Items are scored on a 4-point Likert scale, with response options relating to how frequent the participant experienced each of the 20 symptoms: (0) rarely or none of the time (less than 1 day); (1) some or a little of the time (1–2 days); (2) occasionally or a moderate amount of time (3–4 days); and (3) most or all of the time (5–7 days). Scores were combined to form an aggregate depression score on a scale of 0 to 60 and those with scores of 16 or above were considered to have depressive symptoms [36], [37]. This threshold has been demonstrated to have acceptable specificity and sensitivity in comparison to clinical interview based measures of depressive disorder [38], [39].

### Covariates

The following covariates were used in analyses: age, marital status (married/cohabiting [reference category], single, divorced/separated and widowed) [40], [41], educational attainment (university, secondary/vocational [reference category], primary or less) [42], [43], material deprivation level score [44], [45] which was based on three questions concerning how often the participants' household did not have enough money to buy food or clothes, or to pay bills such as housing, heating and electricity, with the possible answers “never”, “rarely”, “sometimes”, “often” or “all the time”, coded as 0–4. These items were summed and then categorised into three groups: low (reference category; scores of 0–1), intermediate (scores of 2–8) and high (scores of 9–12). Smoking status (never smoked [reference category], current smoker, ex-smoker) was also controlled for [40], [46].

### Statistical Analysis

Logistic regression was used to estimate odds ratios (ORs) and associated 95% confidence intervals (CIs) for depressive symptoms by binge drinking dose/frequency, annual volume of alcohol intake as well as problem drinking. Separate models were fitted for each drinking measure as including all drinking components in the same model would likely represent over-adjustment [47] and therefore bias the effect estimates (estimates from models including all drinking variables simultaneously are made available for interested readers in *Information S2*).

To compare the consistency of associations across countries, we tested for interactions between drinking variables and cohort; we found p-values <0.01 in all interaction tests. Models were further stratified by gender (frequency/dose variables differed by sex). Within all centres, we present two models: model 1 was adjusted for age only, and model 2 was adjusted for all covariates.

All models were fitted using Stata 13.1 [48]. An alpha level of 0.05 was considered statistically significant for all analyses.

## Results

### Sample composition

Table 1 outlines descriptive statistics by country and gender. Briefly, among men, problem drinking was more common in Russia followed by Poland and the Czech Republic. Binge drinking in general was also more common in Russian men with almost 30% of respondents reporting binge drinking on a weekly basis compared to approximately 18% of Czech Republic men and 7% of Polish men. Men from the Czech Republic and Russia consumed a higher volume of alcohol annually than those from Poland. A greater proportion of Polish men abstained from alcohol in the previous year, followed by Russia and the Czech Republic. Depressive symptoms were also more common in Polish men.

**Table 1:** Descriptive information by country and sex.

	Czech				Russia				Poland			
	Men		Women		Men		Women		Men		Women	
	N	%	N	%	N	%	N	%	N	%	N	%
**Depressive symptoms**												
No	3052	86.2	3092	76.2	2636	85.0	2526	66.4	3789	79.3	3412	67.0
Yes	489	13.8	968	23.8	465	15.0	1281	33.6	988	20.7	1683	33.0
**Drinking session dose/frequency**												
Abstainer	227	6.4	738	18.2	418	13.5	620	16.3	1090	22.8	2439	47.9
Always <60 g in a single sitting	887	25.1	2091	51.5	444	14.3	2451	64.4	1511	31.6	2069	40.6
60+g occasionally	1091	30.8	837	20.6	579	18.7	464	12.2	1215	25.4	443	8.7
60–99 g monthly	450	12.7	226	5.6	358	11.5	173	4.5	361	7.6	87	1.7
100–139 g† monthly	159	4.5	78	1.9	205	6.6	32	0.8	131	2.7	26	0.5
140+g monthly	107	3.0	––	––	170	5.5	––	––	152	3.2	––	––
60–99 g weekly	399	11.3	56	1.4	462	14.9	53	1.4	186	3.9	23	0.5
100–139 g† weekly	121	3.4	34	0.8	259	8.4	14	0.4	62	1.3	8	0.2
140+g weekly	100	2.8	––	––	206	6.6	––	––	69	1.4	––	––
**Annual drinking volume**												
Abstainer	227	6.4	738	18.2	418	13.5	620	16.3	1090	22.8	2439	47.9
< = 240 g	389	11.0	1165	28.7	137	4.4	1274	33.5	565	11.8	1185	23.3
1^st^ tertile	753	21.3	388	9.6	757	24.4	933	24.5	1315	27.5	423	8.3
2^nd^ tertile	888	25.1	669	16.5	820	26.4	581	15.3	1140	23.9	589	11.6
3^rd^ tertile	1284	36.3	1100	27.1	969	31.3	399	10.5	667	14.0	458	9.00
**CAGE score**												
<2	2940	90.0	3193	97.3	2066	77.0	3126	98.1	3198	88.2	2556	98.3
2+	326	10.0	88	2.7	617	23.0	61	1.9	430	11.8	45	1.7
Age (mean, SD)	3541	58.4 (7.2)	4060	57.7 (7.1)	3101	58.1 (7.0)	3807	57.9 (7.1)	4777	57.9 (7.0)	5095	57.5 (7.0)
**Marital status**												
Married/cohabiting	2976	84.0	2776	68.4	2743	88.5	2308	60.6	4139	86.6	3399	66.7
Single	105	3.0	98	2.4	70	2.3	172	4.5	197	4.1	357	7.0
Divorced/separated	347	9.8	615	15.1	166	5.4	549	14.4	261	5.5	465	9.1
Widowed	113	3.2	571	14.1	122	3.9	778	20.4	180	3.8	874	17.2
**Educational attainment**												
Primary or less	205	5.8	725	17.9	182	5.9	227	6.0	464	9.7	718	14.1
Secondary/vocational	2667	75.3	2900	71.4	1870	60.3	2524	66.3	2920	61.1	3025	59.4
University	669	18.9	435	10.7	1049	33.8	1056	27.7	1393	29.2	1352	26.5
**Smoking status**												
Never	1121	31.7	2214	54.5	759	24.5	3225	84.7	1325	27.7	2602	51.1
Current	1055	29.8	962	23.7	1505	48.5	392	10.3	1717	35.9	1442	28.3
Former	1365	38.6	884	21.8	837	27.0	190	5.0	1735	36.3	1051	20.6
**Material deprivation**												
Low	2247	66.3	2396	59.0	1462	47.2	961	25.2	2854	59.7	2544	49.9
Intermediate	1140	32.2	1580	38.9	1493	48.2	2498	65.6	1680	35.2	2203	43.2
High	54	1.5	84	2.1	146	4.7	348	9.1	243	5.09	348	6.8

† The figures presented for the category of 100–139 g in a single sitting for women also contain those drinking 140+ g; see methods section.

Some percentage totals may exceed 100 due to rounding.

Among women, abstaining rates were high in Poland doubling the rate of both Russia and the Czech Republic. Problem drinking was highest for women from the Czech Republic followed by those from Russia and Poland. A greater proportion of those from the Czech Republic and Russia engaged in binge drinking on a monthly or weekly basis, although the behaviour was not common in any country (especially heavy binge drinking). Women from the Czech Republic had a higher annual intake of alcohol than women from Russia and Poland. Depressive symptoms were more common amongst women; almost a quarter of women from the Czech Republic and, 34 and 33% of women from Russia and Poland were identified as having depressive symptoms.

### Regression estimates

For men (Table 2), in the Czech Republic significant increased odds of depressive symptoms were observed for those drinking 140+g on a weekly basis (OR 1.92, CI 1.16–3.17) in comparison to those always drinking <60 g in a single sitting. In Poland, binge drinking on a weekly basis was associated with increased odds of depressive symptoms irrespective of the amount consumed in a single session (however upon adjustment for confounding factors the effects were attenuated). Russian men who consumed between 100 to 139 g of ethanol in a single sitting on a monthly basis had lower odds of depressive symptoms (OR 0.53, CI 0.31–0.89) than those who always drank <60 g in a single sitting and this reduced odds was maintained after adjustment for confounding factors (OR 0.47, CI 0.28–0.81). Men who abstained from alcohol in the previous 12 months also had higher odds of depressive symptoms in the Czech Republic (adjusted OR 1.55, CI 1.05–2.28) as well as Poland (adjusted OR 1.59, CI 1.31–1.94). In terms of annual volume of alcohol consumed, those who abstained from alcohol had consistently higher odds of depressive symptoms than those in the second tertile of consumption (after full adjustment this effect was attenuated and became non-significant in the Czech Republic but the overall OR continued to indicate increased odds). In Russia and Poland, those drinking ≤240 g of alcohol annually had increased odds of depressive symptoms when compared to those in the second tertile of consumption, as did those belonging to the first tertile of consumption in Russia (adjusted OR 1.39, CI 1.04, 1.87). Only in Poland was there an increased risk in the third tertile of annual alcohol consumption (adjusted OR 1.42, CI 1.11, 1.81)

**Table 2:** Odds ratios (95% confidence intervals) for depressive symptoms by binge drinking dose/frequency, annual drinking volume and problem drinking by country in men.

	Czech (N = 3541)		Russia (N = 3101)		Poland (N = 4777)	
	Age adjusted	Fully adjusted‡	Age adjusted	Fully adjusted	Age adjusted	Fully adjusted
**Binge drinking dose/frequency**						
Abstainer	1.73** [1.19,2.51]	1.55* [1.05,2.28]	1.12 [0.79,1.59]	1.03 [0.71,1.48]	1.67*** [1.38,2.01]	1.59*** [1.31,1.94]
Always <60 g in a single sitting	1.00 (ref.)	1.00 (ref.)	1.00 (ref.)	1.00 (ref.)	1.00 (ref.)	1.00 (ref.)
60+g occasionally	0.88 [0.68,1.16]	0.98 [0.74,1.28]	0.77 [0.55,1.08]	0.77 [0.55,1.10]	0.88 [0.72,1.08]	0.88 [0.72,1.08]
60–99 g monthly	0.92 [0.66,1.30]	1.01 [0.71,1.44]	0.86 [0.59,1.25]	0.81 [0.55,1.21]	0.98 [0.73,1.31]	0.92 [0.68,1.25]
100–139 g monthly	0.91 [0.55,1.51]	1.01 [0.60,1.69]	0.53* [0.31,0.89]	0.47** [0.28,0.81]	1.06 [0.68,1.67]	0.97 [0.61,1.55]
140+g monthly	1.30 [0.76,2.23]	1.18 [0.68,2.05]	1.18 [0.75,1.87]	1.02 [0.63,1.66]	0.78 [0.50,1.24]	0.67 [0.42,1.07]
60–99 g weekly	0.84 [0.59,1.21]	0.83 [0.57,1.21]	0.66* [0.45,0.97]	0.68 [0.46,1.00]	1.44* [1.01,2.06]	1.38 [0.95,2.01]
100–139 g weekly	0.92 [0.52,1.62]	0.89 [0.50,1.59]	0.78 [0.51,1.21]	0.77 [0.49,1.21]	2.36** [1.38,4.04]	1.71 [0.96,3.04]
140+g weekly	1.92* [1.16,3.17]	1.67 [1.00,2.81]	0.98 [0.62,1.53]	0.83 [0.52,1.32]	1.93* [1.14,3.26]	1.53 [0.88,2.65]
**Annual drinking volume**						
Abstainer	1.72** [1.18,2.50]	1.43 [0.97,2.11]	1.64** [1.18,2.26]	1.55** [1.11,2.17]	1.89*** [1.53,2.32]	1.88*** [1.52,2.34]
≤240 g	1.08 [0.76,1.52]	0.99 [0.70,1.41]	1.68* [1.05,2.69]	1.61 [0.99,2.63]	1.25 [0.96,1.62]	1.32* [1.01,1.72]
1^st^ tertile	0.97 [0.73,1.28]	0.97 [0.73,1.30]	1.36* [1.02,1.81]	1.39* [1.04,1.87]	1.04 [0.84,1.28]	1.08 [0.87,1.34]
2^nd^ tertile	1.00 (ref.)	1.00 (ref.)	1.00 (ref.)	1.00 (ref.)	1.00 (ref.)	1.00 (ref.)
3^rd^ tertile	0.87 [0.68,1.12]	0.83 [0.64,1.07]	1.18 [0.90,1.56]	1.17 [0.88,1.55]	1.48** [1.17,1.88]	1.42** [1.11,1.81]
**Problem drinking** †						
No	1.00 (ref.)	1.00 (ref.)	1.00 (ref.)	1.00 (ref.)	1.00 (ref.)	1.00 (ref.)
Yes	2.18*** [1.65,2.89]	2.09*** [1.56,2.78]	1.82*** [1.44,2.31]	1.66*** [1.29,2.13]	3.17*** [2.55,3.94]	2.76*** [2.20,3.48]

*** p<0.001;

** p<0.01;

* p<0.05.

† Models concerning problem drinking (CAGE score 2+) are restricted to those who consumed alcohol in the previous 12 months (Czech N = 3266, Russia N = 2683, Poland N = 3828).

‡ Full adjustment = age, marital status, educational attainment, material deprivation level and smoking status.

For women (Table 3), those who abstained from alcohol in the previous 12 months had increased odds of depressive symptoms when compared to those always drinking <60 g in a single sitting in the Czech Republic (adjusted OR 1.30, CI 1.07–1.59) and Poland (adjusted OR 1.35, CI 1.18, 1.54). In the Czech Republic, women consuming 100 g+ on a weekly basis had increased odds of depressive symptoms (OR 2.45, CI 1.23–4.90) however this was attenuated after controlling for confounding factors (OR 1.93, CI 0.94–3.96). In both Russia (adjusted OR 3.31, CI 1.52–6.55) and Poland (adjusted OR 2.84, CI 1.26–6.41) women consuming 100+g on a monthly basis had increased odds of depressive symptoms in comparison to those who always drank <60 g in a single sitting. When looking at differences in the odds of depressive symptoms by total volume of alcohol consumed in the previous year, abstainers in all countries had higher odds than those in the second tertile of alcohol intake. In Russia, those with an intake of ≤240 g had higher odds of depressive symptoms (adjusted OR 1.31, CI 1.05–1.64).

**Table 3:** Odds ratios (95% confidence intervals) for depressive symptoms by binge drinking dose/frequency, annual drinking volume and problem drinking by country in women.

	Czech (N = 4060)		Russia (N = 3807)		Poland (N = 5095)	
	Age adjusted	Fully adjusted‡	Age adjusted	Fully adjusted	Age adjusted	Fully adjusted
**Binge drinking dose/frequency**						
Abstainer	1.48*** [1.22,1.79]	1.30** [1.07,1.59]	1.19 [0.99,1.44]	1.14 [0.94,1.37]	1.43*** [1.25,1.62]	1.35*** [1.18,1.54]
Always <60 g in a single sitting	1.00 (ref.)	1.00 (ref.)	1.00 (ref.)	1.00 (ref.)	1.00 (ref.)	1.00 (ref.)
60+g occasionally	1.06 [0.88,1.29]	1.10 [0.90,1.35]	0.91 [0.73,1.13]	0.85 [0.68,1.07]	1.24 [1.00,1.55]	1.17 [0.93,1.47]
60–99 g monthly	0.80 [0.56,1.14]	0.81 [0.57,1.17]	1.02 [0.73,1.43]	1.01 [0.72,1.41]	0.99 [0.62,1.59]	0.90 [0.54,1.48]
100+g monthly	1.46 [0.89,2.41]	1.38 [0.82,2.32]	3.31** [1.62,6.77]	3.16** [1.52,6.55]	2.85** [1.31,6.19]	2.84* [1.26,6.41]
60–99 g weekly	1.06 [0.56,1.99]	1.09 [0.57,2.08]	1.22 [0.69,2.16]	1.26 [0.71,2.25]	0.86 [0.34,2.20]	0.81 [0.31,2.13]
100+g weekly	2.45* [1.23,4.90]	1.93 [0.94,3.96]	1.30 [0.43,3.90]	0.96 [0.31,2.97]	1.47 [0.35,6.17]	1.53 [0.34,6.86]
**Annual drinking volume**						
Abstainer	1.52*** [1.19,1.95]	1.32* [1.02,1.70]	1.39** [1.09,1.78]	1.37* [1.06,1.76]	1.56*** [1.28,1.91]	1.48*** [1.19,1.83]
≤240 g annually	1.14 [0.90,1.43]	1.11 [0.88,1.41]	1.31* [1.06,1.63]	1.31* [1.05,1.64]	1.22 [0.98,1.52]	1.18 [0.94,1.49]
1^st^ tertile	1.05 [0.78,1.42]	0.97 [0.71,1.32]	1.04 [0.83,1.30]	1.10 [0.87,1.38]	1.01 [0.76,1.34]	1.00 [0.74,1.33]
2^nd^ tertile	1.00 (ref.)	1.00 (ref.)	1.00 (ref.)	1.00 (ref.)	1.00 (ref.)	1.00 (ref.)
3^rd^ tertile	0.98 [0.78,1.24]	1.00 [0.79,1.27]	1.25 [0.95,1.64]	1.28 [0.96,1.69]	1.29 [0.99,1.68]	1.30 [0.99,1.72]
**Problem drinking** †						
No	1.00 (ref.)	1.00 (ref.)	1.00 (ref.)	1.00 (ref.)	1.00 (ref.)	1.00 (ref.)
Yes	2.36*** [1.52,3.66]	2.28*** [1.45,3.59]	2.51*** [1.51,4.18]	2.38** [1.41,4.03]	3.89*** [2.12,7.16]	3.37*** [1.77,6.42]

*** p<0.001;

** p<0.01;

* p<0.05.

† Models concerning problem drinking (CAGE score 2+) are restricted to those who consumed alcohol in the previous 12 months (Czech N = 3541, Russia N = 3101, Poland N = 4777).

‡ Full adjustment = age, marital status, educational attainment, material deprivation level and smoking status.

Problem drinking was consistently associated with increased odds of depressive symptoms across all countries in both men (Table 2) and women (Table 3).

When stratifying the abstainer group into former drinkers and life-long abstainers (Table 4) in the Russian cohort, neither abstainer category had increased odds of depressive symptoms compared to those always drinking <60 g in a single sitting for men, while for women, former drinkers had increased odds (adjusted OR 1.37, CI 1.09–1.72) but not lifelong abstainers. Former drinkers had higher odds of depressive symptoms in all models relating to annual alcohol intake in comparison to those in the second tertile of consumption (adjusted ORs of 1.55, CI 1.10, 2.17 and 1.64, CI 1.23, 2.17 for men and women respectively).

**Table 4:** Odds ratios (95% confidence intervals) for former drinkers and lifelong abstainers on depressive symptoms in the Russian HAPIEE cohort.

	Men			Women		
	N	Age adjusted	Fully adjusted‡	N	Age adjusted	Fully adjusted
**Binge drinking dose/frequency**						
Always <60 g in a single sitting	––	1.00 [1.00,1.00]	1.00 [1.00,1.00]	––	1.00 [1.00,1.00]	1.00 [1.00,1.00]
Former drinker	388	1.12 [0.78,1.59]	1.02 [0.71,1.48]	369	1.41** [1.13,1.77]	1.37** [1.09,1.72]
Lifelong abstainer	30	1.17 [0.46,2.98]	1.07 [0.41,2.80]	251	0.92 [0.70,1.22]	0.86 [0.65,1.14]
**Annual drinking volume**						
2^nd^ tertile	––	1.00 [1.00,1.00]	1.00 [1.00,1.00]	––	1.00 [1.00,1.00]	1.00 [1.00,1.00]
Former drinker	388	1.63** [1.17, 2.27]	1.55** [1.10, 2.17]	369	1.65*** [1.25, 2.18]	1.64*** [1.23, 2.17]
Lifelong abstainer	30	1.72 [0.69, 4.33]	1.63 [0.63, 4.23]	251	1.07 [0.78, 1.48]	1.03 [0.74, 1.43]

*** p<0.001;

** p<0.01;

* p<0.05.

‡ Full adjustment = age, marital status, educational attainment, material deprivation level and smoking status.

## Discussion

### Summary of findings

In this large population based study in Eastern Europe, we found that problem drinking was consistently associated with an increased prevalence of depressive symptoms in all populations and both sexes. With binge drinking, the results were slightly less consistent. In men, frequent heavy binge drinking (140+ g in a sitting at least once a week) was associated with increased odds of depressive symptoms in the Czech Republic and Poland. Interestingly, Russian men who drank 100–139 g of alcohol in a single sitting on a monthly basis had lower odds of depressive symptoms than those always drinking less than 60 g in a single sitting. Among women, there was a tendency for those who engaged in heavy binge drinking (100+g in a single sitting) to have higher odds of depressive symptoms but the odds ratios were not statistically significant in the case of at least weekly binge drinking.

Abstaining from alcohol was also associated with increased odds of depressive symptoms in comparison to moderate drinking except in Russia; however, compared to the second tertile of annual alcohol consumption, abstainers had consistently higher odds of depressive symptoms. Analyses attempting to disentangle lifelong non-drinkers from former drinkers typically revealed significantly increased odds of depressive symptoms in former drinkers.

### Interpretation and comparison to other work

These findings corroborate previous studies in Central and Eastern Europe which have found binge drinking to be associated with increased rates of depressive symptoms [22], including earlier analyses of the HAPIEE data [15]. However, the models used previously [15] differ substantially from those used here. The previous analyses used a relatively low dose of alcohol (<20 g, including abstainers) as the reference category (in comparison to the referent group used in this study, i.e. always consuming <60 g in a single sitting and excluding abstainers) and they used the mean dose of alcohol in a single drinking session as the main exposure (using a maximum dose of ≥80 g). While the previous analyses found increased odds of depressive symptoms in binge drinkers, the previous definition did not take into account the frequency of binge drinking or the graded increase in the amount of alcohol consumed in a binge drinking session (i.e. different doses above 60 g). When the intensity and frequency of binge drinking are accounted for, as in our analyses, it appears that both elements are important in terms of the association with depressive symptoms.

An unexpected finding was that Russian men who consumed 100–139 g in a single sitting at least monthly had lower odds of depressive symptoms than those who always consumed less than 60 g in a drinking occasion. A possible explanation for this finding is that those who drank heavily did so with friends and therefore social participation served as a protective factor against depression, whilst also increasing the opportunity to drink at hazardous levels [49], [50]. However, given that this result was not replicated in the Czech Republic or Polish cohorts (or in women) appropriate caution should be applied in the interpretation this this finding.

To our knowledge only one other study has examined abstaining from alcohol as a risk factor for poor mental health in Eastern Europe, focussing on Russian men [51]. Our findings are generally consistent with that study in that we found abstaining was not associated with greater odds of depressive symptoms in Russia when looking at drinking pattern (effect estimates indicated poorer mental health for abstaining Russians in both studies but these effects did not reach statistical significance) but in analyses of the association between annual drinking volume and depressive symptoms, Russians who abstained from alcohol actually had significantly increased odds of depression.

In our data, problem drinkers had consistently increased odds of depressive symptoms. This finding is in line with previous findings on the topic of depression and alcohol consumption in Eastern Europe [22], [23]. In studies elsewhere, problem drinking has been demonstrated to increase the risk of prevalent [9], [22], [52]–[56] and incident [6]–[8], [57] depression independent of the amount of alcohol a person consumes or the manner in which that they consume it [21], [58].

### Strengths and weaknesses

Specific strengths of this analysis include the large sample size of each country – providing relatively good statistical power (with certain exceptions outlined below). Furthermore, standardised internationally validated tools were used to collect information on individual drinking episodes and depressive symptoms. Data from the graduated frequency questionnaire were then used to create a variable which took into account both dose and frequency of binge drinking and derive total annual alcohol intake.

There are, however, also several shortcomings. First, these analyses are based on cross-sectional data. It is plausible that the relationship would differ over time and examining the association over multiple occasions may provide additional aetiological insights into the possibly bi-directional relationship between alcohol consumption and depressive symptoms [5], [59], [60] (that is, depressive symptoms may cause people to drink in binges, and drinking in binges may exacerbate depressive symptoms). Therefore further work using longitudinal data is warranted.

Another limitation is that in some of our statistical models, abstainers had to be grouped into a single aggregate category of past year non-drinkers, although the reasons for present/recent non-drinking are likely to be heterogeneous. Many other investigators have pointed out the weaknesses of using an aggregate group of abstainers [26], [27], [61] as a comparison group for moderate (and other) drinkers. There are valid concerns that moderate drinking may inadvertently be attributed as having beneficial effects compared to non-drinking when the reality may be that this difference is due to residual confounding [62] or failure to distinguish former drinkers and occasional drinkers from the lifelong abstaining group [27], [63]. In our study we were able to stratify the abstainer group into former drinkers and lifelong abstainers in Russia (Table 4). We found that there were increased odds of depressive symptoms amongst former drinkers, supporting the argument that the increased odds of depressive symptoms among abstainers may largely be driven by former drinkers as previously reported elsewhere [64].

A third limitation is that the frequency of consuming <60 g in a single sitting was not taken into account. The reference group used in these analyses may therefore contain a number of occasional or, conversely, frequent drinkers which may bias the relationship observed [27]. However, additional analyses did not detect a significant association between depressive symptoms and drinking frequency in this group (with the exception of Russian women, where those drinking very infrequently (only 1–5 times per year) had increased odds of depression; data not presented). It is also possible that the reference group contains some individuals who have reduced their alcohol consumption over time due to ill-health [65], [66]. There was no information available to account for this possibility at baseline, and interpretation of the above findings should be made with this in mind.

Fourth, there may also be concerns about the small number of female binge drinkers identified in the study, especially categories relating to heavy and frequent binge drinking. The odds ratios for female drinkers have wide confidence intervals and should be interpreted with appropriate caution.

A fifth limitation relates to both alcohol and depressive symptom measures being based on self-reported data. Self-report measures of alcohol consumption have been consistently demonstrated to provide inaccurate estimates of consumption [67], [68] and self-report measures of depressive symptoms are not (necessarily) equivalent to clinical diagnoses [69].

Sixth, participants were aged between 45 and 69 years of age. It is plausible that the same relationship might not be present in younger or older populations. Studies examining the relationship between alcohol use disorders and major depression earlier in the life-course also find a consistent relationship between problematic drinking and depression [7] suggesting that the association between these two measures might be consistent across the life-course. However, drinking pattern is known to fluctuate over time [70] and hazardous drinking declines with age [71] – this may explain why we do not see a consistent picture with respect to the association between binge drinking or annual volume of alcohol consumed and depressive symptoms in our sample. Analyses focussed on life-course patterns of alcohol intake in relation to depressive symptomology may provide important additional information, such as whether there are periods in the life-course whereby binge drinking is most commonly linked with depressive symptoms or if the relationship between drinking pattern and depressive symptoms is best explained by accumulated exposure of life-time drinking. Such hypotheses strengthen the argument that longitudinal work on this topic is warranted.

Finally, the rates of abstention in Poland were higher than in other populations in HAPIEE and than what we would expect from population level surveys [4]. Almost 35% of the Polish sample reported being non-drinkers at baseline. The reason for such a high rate is believed to be related to the Polish questionnaire containing a filter question which allowed for participants to skip the alcohol related questions if they indicated being non-drinkers in the last 12 months. However, the associations of abstention with depressive symptoms in Polish subjects were similar to Czech participants, as would be expected from the geographical and cultural closeness of these two populations. This suggests that the filter questions did not introduce a major bias.

## Conclusions

Our findings indicate that dose of alcohol consumed in a binge drinking session and the frequency of doing so may contribute to the burden of depression in Eastern Europe but problem drinking was the alcohol variable consistently associated with increased odds of depressive symptoms in all countries, indicating that it is likely to be a useful predictor of depression in Eastern Europe. It is possible that the increased odds of depressive symptoms observed amongst non-drinkers is driven by former drinkers. Future work will however need to investigate whether this relationship holds when predicting depressive symptoms prospectively using a longitudinal design.

## Supporting Information

Information S1 Estimates from models relating the frequency of drinking >60, 100 and 140 g in the past year to depressive symptoms.
(PDF)

Information S2 Estimates from models whereby binge drinking dose/frequency, annual drinking volume and problem drinking are simultaneously included in the same model.
(PDF)
