# Supplementary material for: Drinking Pattern, Abstention and Problem Drinking as Risk Factors for Depressive Symptoms: Evidence from Three Urban Eastern European Populations
Source: PLoS One. 2014 Aug 13;9(8):e104384. doi: 10.1371/journal.pone.0104384 (PMC4131916; doi:10.1371/journal.pone.0104384)
Supplement: Information S1 — Estimates from models relating the frequency of drinking >60, 100 and 140 g in the past year to depressive symptoms. (PDF) [file pone.0104384.s001.pdf]

Table of Contents for Supporting Information 1

Supporting Information 1 contains estimates from models relating the frequency of drinking > 60, 100 and 140 g in the past year to depressive symptoms.

In these models, the reference group are those who always drank < 60 g in a single sitting. As outlined in the main manuscript at doses of 100+ and 140+ g per occasion an additional category was included to represent those who drank above 60+ g but always less than *X* g per occasion (e.g. in the case of 100+ g, if someone drank 80g on a weekly basis they would fall in the category of “Never > 100 g per occasion”).

1. Table S1.1 - Descriptive information regarding frequency of > 60, 100 and 140 g in a single drinking occasion by country
2. Table S1.2 - Odds ratios (95% confidence intervals) for depressive symptoms by frequency of consuming > 60, 100 and 140 g in a single drinking occasion by country in men
3. Table S1.3 – Odds ratios (95% confidence intervals) for depressive symptoms by frequency of consuming > 60, 100 and 140 g in a single drinking occasion by country in women

Table S1.1 – Descriptive information regarding frequency of > 60, 100 and 140 g in a single drinking occasion by country

	Czech				Russia				Poland			
	**Men**		**Women**		**Men**		**Women**		**Men**		**Women**	
	**N**	**%**	**N**	**%**	**N**	**%**	**N**	**%**	**N**	**%**	**N**	**%**
**Frequency of X amount**												
**> 60g**												
Abstainer	227	6.4	738	18.2	418	13.5	620	16.3	1090	22.8	2439	47.9
Always < 60 g in a single sitting	887	25	2091	51.5	444	14.3	2451	64.4	1511	31.6	2069	40.6
1-11 times per year	1091	30.8	837	20.6	579	18.7	464	12.2	1215	25.4	443	8.7
1-3 times per month	716	20.2	304	7.5	733	23.6	205	5.4	644	13.5	113	2.2
Weekly	620	17.5	90	2.2	927	29.9	67	1.8	317	6.6	31	0.6
**> 100g**												
Abstainer	227	6.4	738	18.2	418	13.5	620	16.3	1090	22.8	2439	47.9
Always < 60 g in a single sitting	887	25	2091	51.5	444	14.3	2451	64.4	1511	31.6	2069	40.6
Never > 100g in a single sitting	863	24.4	724	17.8	814	26.2	609	16	805	16.9	401	7.9
1-11 times per year	924	26.1	384	9.5	486	15.7	79	2.1	914	19.1	148	2.9
1-3 times per month	419	11.8	89	2.2	474	15.3	34	0.9	326	6.8	30	0.6
Weekly	221	6.2	34	0.8	465	15	14	0.4	131	2.7	8	0.2
**> 140g**												
Abstainer	227	6.4	738	18.2	418	13.5	620	16.3	1090	22.8	2439	47.9
Always < 60 g in a single sitting	887	25	2091	51.5	444	14.3	2451	64.4	1511	31.6	2069	40.6
Never > 140g in a single sitting	1551	43.8	996	24.5	1389	44.8	697	18.3	1389	29.1	507	10
1-11 times per year	583	16.5	166	4.1	400	12.9	27	0.7	530	11.1	62	1.2
1-3 times per month	193	5.5	44	1.1	244	7.9	10	0.3	188	3.9	14	0.3
Weekly	100	2.8	25	0.6	206	6.6	2	0.1	69	1.4	4	0.1

Some percentage totals may not equal 100 due to rounding

Table S1.2 - Odds ratios (95% confidence intervals) for depressive symptoms by frequency of consuming > 60, 100 and 140 g in a single drinking occasion by country in men

	**Czech (N=3541)**		**Russia (N=3101)**		**Poland (N=4777)**	
	Age adjusted	Fully adjusted	Age adjusted	Fully adjusted	Age adjusted	Fully adjusted
**Frequency of X amount**						
**> 60g**						
Abstainer	1.73** [1.19,2.51]	1.54* [1.05,2.27]	1.12 [0.79,1.58]	1.02 [0.71,1.47]	1.67*** [1.38,2.01]	1.59*** [1.31,1.94]
Always < 60 g in a single sitting	1.00 (ref.)	1.00 (ref.)	1.00 (ref.)	1.00 (ref.)	1.00 (ref.)	1.00 (ref.)
1-11 times per year	0.88 [0.68,1.16]	0.98 [0.74,1.29]	0.77 [0.55,1.08]	0.77 [0.55,1.10]	0.88 [0.72,1.08]	0.88 [0.72,1.08]
1-3 times per month	0.97 [0.72,1.31]	1.04 [0.77,1.41]	0.83 [0.60,1.14]	0.75 [0.54,1.05]	0.95 [0.74,1.20]	0.86 [0.67,1.11]
Weekly	1.01 [0.75,1.37]	0.97 [0.71,1.34]	0.76 [0.56,1.04]	0.74 [0.53,1.02]	1.71*** [1.29,2.25]	1.48** [1.11,1.99]
**> 100g**						
Abstainer	1.72** [1.19,2.51]	1.54* [1.05,2.27]	1.12 [0.79,1.58]	1.02 [0.71,1.47]	1.67*** [1.38,2.01]	1.60*** [1.31,1.94]
Always < 60 g in a single sitting	1.00 (ref.)	1.00 (ref.)	1.00 (ref.)	1.00 (ref.)	1.00 (ref.)	1.00 (ref.)
Never > 100g in a single sitting	1.11 [0.84,1.45]	1.20 [0.91,1.58]	0.83 [0.61,1.14]	0.83 [0.60,1.15]	0.91 [0.73,1.14]	0.89 [0.71,1.13]
1-11 times per year	0.72* [0.53,0.96]	0.78 [0.58,1.06]	0.67* [0.47,0.97]	0.68* [0.46,0.99]	0.93 [0.75,1.15]	0.92 [0.73,1.15]
1-3 times per month	0.89 [0.62,1.26]	0.87 [0.61,1.26]	0.74 [0.51,1.06]	0.65* [0.45,0.96]	1.12 [0.83,1.50]	0.98 [0.71,1.34]
Weekly	1.32 [0.88,1.96]	1.21 [0.80,1.83]	0.87 [0.60,1.24]	0.79 [0.54,1.15]	2.13*** [1.44,3.13]	1.62* [1.07,2.44]
**> 140g**						
Abstainer	1.73** [1.19,2.51]	1.55* [1.05,2.28]	1.12 [0.79,1.58]	1.03 [0.71,1.48]	1.67*** [1.38,2.01]	1.59*** [1.31,1.94]
Always < 60 g in a single sitting	1.00 (ref.)	1.00 (ref.)	1.00 (ref.)	1.00 (ref.)	1.00 (ref.)	1.00 (ref.)
Never > 140g in a single sitting	0.92 [0.72,1.18]	1.00 [0.78,1.29]	0.80 [0.60,1.07]	0.79 [0.58,1.06]	0.93 [0.77,1.13]	0.91 [0.75,1.11]
1-11 times per year	0.72 [0.52,1.01]	0.76 [0.54,1.07]	0.48*** [0.31,0.74]	0.48** [0.31,0.75]	1.06 [0.82,1.36]	1.01 [0.78,1.31]
1-3 times per month	1.37 [0.90,2.08]	1.25 [0.81,1.93]	1.07 [0.70,1.62]	0.95 [0.61,1.47]	1.13 [0.78,1.65]	0.93 [0.63,1.37]
Weekly	1.91* [1.16,3.16]	1.67 [0.99,2.80]	0.98 [0.62,1.53]	0.83 [0.52,1.32]	1.93* [1.14,3.27]	1.53 [0.88,2.65]

*** p < 0.001; ** p < 0.01; * p < 0.05

‡ Full adjustment = age, marital status, educational attainment, material deprivation level and smoking status

Table S1.3 - Odds ratios (95% confidence intervals) for depressive symptoms by frequency of consuming > 60, 100 and 140 g in a single drinking occasion by country in women

	**Czech (N=4060)**		**Russia (N=3807)**		**Poland (N=5095)**	
	Age adjusted	Fully adjusted	Age adjusted	Fully adjusted	Age adjusted	Fully adjusted
**Frequency of X amount**						
**> 60g**						
Abstainer	1.48*** [1.22,1.79]	1.30** [1.07,1.58]	1.20 [0.99,1.44]	1.14 [0.94,1.37]	1.43*** [1.26,1.62]	1.35*** [1.18,1.54]
Always < 60 g in a single sitting	1.00 (ref.)	1.00 (ref.)	1.00 (ref.)	1.00 (ref.)	1.00 (ref.)	1.00 (ref.)
1-11 times per year	1.06 [0.88,1.29]	1.10 [0.90,1.35]	0.91 [0.73,1.13]	0.85 [0.68,1.06]	1.24 [1.00,1.55]	1.17 [0.93,1.47]
1-3 times per month	0.95 [0.71,1.28]	0.95 [0.70,1.29]	1.25 [0.92,1.68]	1.21 [0.89,1.65]	1.29 [0.86,1.92]	1.20 [0.79,1.83]
Weekly	1.50 [0.94,2.39]	1.38 [0.85,2.24]	1.24 [0.74,2.06]	1.18 [0.70,1.99]	1.00 [0.46,2.18]	0.96 [0.43,2.17]
**> 100g**						
Abstainer	1.47*** [1.22,1.78]	1.30** [1.07,1.58]	1.20 [0.99,1.44]	1.14 [0.94,1.38]	1.43*** [1.25,1.62]	1.35*** [1.17,1.54]
Always < 60 g in a single sitting	1.00 (ref.)	1.00 (ref.)	1.00 (ref.)	1.00 (ref.)	1.00 (ref.)	1.00 (ref.)
Never > 100g in a single sitting	1.29* [1.00,1.66]	1.32* [1.02,1.72]	1.03 [0.63,1.67]	0.98 [0.59,1.60]	1.53* [1.08,2.16]	1.48* [1.03,2.12]
1-11 times per year	1.37 [0.85,2.21]	1.30 [0.79,2.13]	3.25*** [1.63,6.49]	3.08** [1.52,6.23]	2.79** [1.35,5.75]	2.83** [1.33,6.03]
1-3 times per month	2.46* [1.23,4.92]	1.95 [0.95,4.00]	1.30 [0.43,3.90]	0.95 [0.31,2.95]	1.47 [0.35,6.18]	1.54 [0.34,6.87]
Weekly	1.47*** [1.22,1.78]	1.30** [1.07,1.58]	1.20 [0.99,1.44]	1.14 [0.94,1.38]	1.43*** [1.25,1.62]	1.35*** [1.17,1.54]
**> 140g**						
Abstainer	1.48*** [1.22,1.79]	1.30** [1.07,1.59]	1.20 [0.99,1.44]	1.14 [0.94,1.38]	1.43*** [1.25,1.62]	1.35*** [1.17,1.54]
Always < 60 g in a single sitting	1.00 (ref.)	1.00 (ref.)	1.00 (ref.)	1.00 (ref.)	1.00 (ref.)	1.00 (ref.)
Never > 140 g in a single sitting	0.96 [0.80,1.16]	1.00 [0.83,1.22]	1.00 [0.83,1.20]	0.95 [0.79,1.15]	1.09 [0.88,1.35]	1.03 [0.83,1.29]
1-11 times per year	1.38 [0.97,1.98]	1.31 [0.91,1.89]	1.33 [0.60,2.92]	1.11 [0.50,2.47]	2.44*** [1.47,4.06]	2.12** [1.25,3.61]
1-3 times per month	1.81 [0.96,3.42]	1.60 [0.83,3.07]	2.22 [0.64,7.72]	1.86 [0.52,6.57]	3.24* [1.12,9.39]	3.12* [1.02,9.50]
Weekly	2.75* [1.24,6.12]	2.18 [0.96,4.95]	2.61 [0.16,41.92]	1.16 [0.07,19.97]	2.47 [0.35,17.60]	2.30 [0.30,17.53]

*** p < 0.001; ** p < 0.01; * p < 0.05

‡ Full adjustment = age, marital status, educational attainment, material deprivation level and smoking status.
