# Supplementary material for: Drinking Pattern, Abstention and Problem Drinking as Risk Factors for Depressive Symptoms: Evidence from Three Urban Eastern European Populations
Source: PLoS One. 2014 Aug 13;9(8):e104384. doi: 10.1371/journal.pone.0104384 (PMC4131916; doi:10.1371/journal.pone.0104384)
Supplement: Information S2 — Estimates from models whereby binge drinking dose/frequency, annual drinking volume and problem drinking are simultaneously included in the same model. (PDF) [file pone.0104384.s002.pdf]

Table of Contents for Supporting Information 2

Supporting Information 2 contains estimates from models whereby binge drinking dose/frequency, annual drinking volume and problem drinking are simultaneously included in the same model.

1. Table S2.1 - Odds ratios (95% confidence intervals) for depressive symptoms when simultaneously including all drinking measures by country in men (drinkers only)
2. Table S2.2 – Odds ratios (95% confidence intervals) for depressive symptoms when simultaneously including all drinking measures by country in women (drinkers only)

Table S1 – Odds ratios (95% confidence intervals) for depressive symptoms when simultaneously including all drinking measures by country in men (drinkers only)

	**Czech (N=3266)**		**Russia (N=2683)**		**Poland (N=3628)**	
	Age adjusted	Fully adjusted	Age adjusted	Fully adjusted	Age adjusted	Fully adjusted
**Binge drinking dose/frequency**						
<60g in a sitting	1.00 (ref.)	1.00 (ref.)	1.00 (ref.)	1.00 (ref.)	1.00 (ref.)	1.00 (ref.)
60+g occasionally	0.93 [0.69,1.26]	1.00 [0.74,1.36]	0.75 [0.52,1.08]	0.75 [0.51,1.09]	0.86 [0.68,1.07]	0.86 [0.69,1.09]
60-99g monthly	1.04 [0.70,1.55]	1.12 [0.75,1.68]	0.92 [0.60,1.40]	0.85 [0.55,1.31]	0.90 [0.64,1.25]	0.86 [0.61,1.21]
100-139g monthly	1.04 [0.59,1.83]	1.10 [0.62,1.96]	0.57 [0.32,1.00]	0.50* [0.28,0.89]	0.86 [0.52,1.41]	0.81 [0.49,1.36]
140+g monthly	1.52 [0.84,2.75]	1.32 [0.71,2.45]	1.14 [0.65,1.99]	0.97 [0.54,1.72]	0.51* [0.30,0.86]	0.45** [0.26,0.77]
60-99g weekly	1.05 [0.66,1.67]	1.03 [0.64,1.66]	0.73 [0.43,1.25]	0.73 [0.42,1.26]	0.98 [0.62,1.55]	0.97 [0.61,1.55]
100-139g weekly	1.15 [0.60,2.20]	1.08 [0.56,2.10]	0.74 [0.40,1.39]	0.72 [0.38,1.37]	1.34 [0.70,2.56]	0.95 [0.48,1.89]
140+g weekly	2.10* [1.14,3.86]	1.86 [1.00,3.48]	0.85 [0.45,1.61]	0.71 [0.36,1.37]	1.09 [0.58,2.04]	0.90 [0.47,1.73]
**Annual drinking volume**						
<= 240g annually	1.13 [0.76,1.67]	1.10 [0.73,1.64]	1.75* [1.01,3.03]	1.59 [0.90,2.81]	1.30 [0.96,1.75]	1.32 [0.97,1.81]
1st tertile of consumption	1.03 [0.77,1.39]	1.04 [0.77,1.41]	1.58** [1.13,2.20]	1.54* [1.10,2.17]	1.07 [0.85,1.34]	1.09 [0.86,1.38]
2nd tertile of consumption	1.00 (ref.)	1.00 (ref.)	1.00 (ref.)	1.00 (ref.)	1.00 (ref.)	1.00 (ref.)
3rd tertile of consumption	0.66* [0.48,0.91]	0.66* [0.48,0.91]	1.05 [0.70,1.59]	1.04 [0.69,1.58]	1.09 [0.81,1.48]	1.14 [0.83,1.56]
**Problem drinking**						
No	1.00 (ref.)	1.00 (ref.)	1.00 (ref.)	1.00 (ref.)	1.00 (ref.)	1.00 (ref.)
Yes	2.34*** [1.72,3.17]	2.30*** [1.69,3.15]	2.17*** [1.65,2.86]	2.01*** [1.51,2.67]	3.38*** [2.65,4.30]	3.06*** [2.38,3.94]

*** p < 0.001; ** p < 0.01; * p < 0.05

Table S2 – Odds ratios (95% confidence intervals) for depressive symptoms when simultaneously including all drinking measures by country in women (drinkers only)

	**Czech (N=3281)**		**Russia (N=3187)**		**Poland (N=2601)**	
	Age adjusted	Fully adjusted	Age adjusted	Fully adjusted	Age adjusted	Fully adjusted
**Binge drinking dose/frequency**						
<60g in a sitting	1.00 (ref.)	1.00 (ref.)	1.00 (ref.)	1.00 (ref.)	1.00 (ref.)	1.00 (ref.)
60+g occasionally	1.20 [0.96,1.50]	1.22 [0.96,1.53]	1.05 [0.81,1.38]	0.96 [0.73,1.26]	1.37* [1.06,1.78]	1.24 [0.95,1.62]
60-99g monthly	0.90 [0.61,1.35]	0.87 [0.58,1.31]	1.23 [0.82,1.83]	1.15 [0.76,1.73]	1.01 [0.59,1.72]	0.83 [0.47,1.45]
100+g monthly	1.65 [0.96,2.85]	1.47 [0.83,2.58]	3.45** [1.57,7.56]	3.08** [1.39,6.85]	2.17 [0.89,5.29]	2.13 [0.86,5.30]
60-99g weekly	1.07 [0.53,2.13]	1.00 [0.49,2.04]	1.34 [0.70,2.57]	1.30 [0.67,2.53]	0.74 [0.27,2.01]	0.66 [0.24,1.83]
100+g weekly	2.31* [1.09,4.87]	1.67 [0.76,3.65]	1.00 [0.30,3.30]	0.68 [0.20,2.33]	0.76 [0.16,3.68]	0.77 [0.15,3.96]
**Annual drinking volume**						
<= 240g annually	1.23 [0.96,1.58]	1.20 [0.93,1.55]	1.41* [1.08,1.84]	1.33* [1.01,1.75]	1.38** [1.08,1.75]	1.29* [1.01,1.66]
1st tertile of consumption	1.05 [0.77,1.43]	0.95 [0.69,1.31]	1.10 [0.84,1.44]	1.11 [0.85,1.46]	1.03 [0.77,1.38]	0.99 [0.73,1.34]
2nd tertile of consumption	1.00 (ref.)	1.00 (ref.)	1.00 (ref.)	1.00 (ref.)	1.00 (ref.)	1.00 (ref.)
3rd tertile of consumption	0.88 [0.68,1.13]	0.93 [0.72,1.21]	0.99 [0.72,1.37]	1.03 [0.74,1.43]	1.14 [0.85,1.54]	1.22 [0.89,1.66]
**Problem drinking**						
No	1.00 (ref.)	1.00 (ref.)	1.00 (ref.)	1.00 (ref.)	1.00 (ref.)	1.00 (ref.)
Yes	2.33*** [1.46,3.73]	2.30*** [1.41,3.73]	2.44** [1.41,4.21]	2.39** [1.37,4.19]	3.76*** [1.97,7.17]	3.20*** [1.62,6.31]

*** p < 0.001; ** p < 0.01; * p < 0.05
